# The tRNA-Derived Fragment tRF-24-V29K9UV3IU Functions as a miRNA-like RNA to Prevent Gastric Cancer Progression by Inhibiting GPR78 Expression

**DOI:** 10.1155/2022/8777697

**Published:** 2022-04-29

**Authors:** Hui Wang, Weikang Huang, Xirui Fan, Xiaoxue He, Sijin Chen, Su Yu, Yan Zhang

**Affiliations:** ^1^Department of Gastroenterology, West China Hospital, Sichuan University, Chengdu, China; ^2^Department of Gastroenterology, The Affiliated YanAn Hospital of Kunming Medical University, Kunming, China

## Abstract

Emerging studies have proved that tRNA-derived fragments (tRFs) play vital roles in tumor metastasis; however, the function of tRFs in gastric cancer (GC) remains largely unclear. We investigated the role of tRF-24-V29K9UV3IU in growth and metastasis of GC using a xenograft mouse model. Differential gene expression downstream of tRF-24-V29K9UV3IU was identified by transcriptome sequencing, and interaction was then verified by a dual luciferase reporter and RNA immunoprecipitation. MKN-45 cells were also used to explore the biological functions of tRF-24-V29K9UV3IU *in vitro*. Here, knockdown of tRF-24-V29K9UV3IU promoted tumor growth and metastasis of GC *in vivo*. The expression of tRF-24-V29K9UV3IU and E-cadherin (epithelial cell marker) was down-regulated in tumors of mice following tRF-24-V29K9UV3IU knockdown, whereas the mesenchymal cell markers N-cadherin and vimentin displayed an opposite trend. Transcriptome sequencing identified 87 differentially expressed genes (DEGs) down-regulated in the tRF-24-V29K9UV3IU-overexpressed groups compared with the control group. Among them, G-protein–coupled receptor 78 (GPR78), the most significantly down-regulated DEG, was also predicted to be a target of tRF-24-V29K9UV3IU. Moreover, tRF-24-V29K9UV3IU could function as a miRNA-like fragment and bind to AGO2 and directly silence GPR78 expression by complementing with the 3′-untranslated region of the GPR78 mRNA. Functionally, overexpression of tRF-24-V29K9UV3IU significantly suppressed proliferation, migration, and invasion and promoted apoptosis of MKN-45 cells, whereas GPR78 attenuated these effects. Therefore, our data suggest that tRF-24-V29K9UV3IU functions as a miRNA-like fragment to suppress GPR78 expression and thus inhibit GC progression. These observations suggest that the tRF-24-V29K9UV3IU/GPR78 axis serves as a potential therapeutic target in GC.

## 1. Introduction

Gastric cancer (GC) ranks second in morbidity and mortality of various cancers in China, only after lung cancer [1]. Most GC patients already demonstrate advanced disease and metastasis at the time of diagnosis; these patients cannot be cured by simple radical surgical resection, which is accompanied by an extremely high recurrence rate [2]. Epidemiological studies have shown that GC is most prevalent in patients aged >55 years [3]. As the aging population of China grows proportionally larger, the social burden caused by GC will also rise; however, clinical treatment of these patients is hampered by the lack of a clear understanding of the molecular mechanisms underlying GC development [4]. Therefore, revealing the molecular mechanisms underlying invasion and metastasis in GC and finding non-invasive, simple, and feasible new biomarkers to prevent and control this malignant disease are of great importance.

tRNA-derived fragments (tRFs) are short non-coding RNAs derived from tRNA, approximately 16–40 nucleotides in length [5], which originate from mitochondrial or nuclear tRNAs [6]. Currently, tRFs are known to be involved in various physiological and pathological processes, such as infectious diseases and tumor formation, and in neurodegenerative diseases [7]. Schimmel reported that the events of tRF production responded to many stresses such as hyperosmotic stress, pro-oncogenic transcription under hypoxia, and regulation of hematopoiesis [8]. The regulatory role of tRFs on tumor progression has been extensively studied. tiRNA-Gly, a kind of 5′-tRNA halve, promotes migration and proliferation of papillary thyroid cancer cell through binding to RBM17 and inducing alternative splicing [9]. The tRF Lys-CTT-010 promoted malignant progression of triple-negative breast cancer through glucose metabolism regulation [10]. tRF-Leu-CAG promotes the proliferation and cell cycle progression of non-small-cell lung cancer cells [11]. These studies suggested that abnormally expressed tRFs are associated with changes in tumor biological function, and it is expected that tRFs can serve as tumor diagnostic or prognostic markers or even tumor therapeutic targets. However, there are few studies on the role of tRFs in GC, and the mechanism by which tRFs regulate biological functions in GC remains unclear.

In our previous study, using small RNA sequencing, we identified that tRF-24-V29K9UV3IU was prominently down-regulated in GC tissues relative to adjacent tissues [12]; the available genomes is *Homo sapiens* (hg19/GRCh37). Moreover, pathway analysis showed that the target genes of tRF-24-V29K9UV3IU were involved in biological processes related to cancer occurrence and metastasis, such as cell adhesion and connection, cell migration, and the cAMP signaling, Wnt signaling, MAPK signaling, and cancer signaling pathways. Therefore, we attempted to further explore the function and regulatory mechanisms of tRF-24-V29K9UV3IU on the invasion and metastasis of GC cells *in vivo* and *in vitro* using a xenograft tumor mouse model and transcriptome sequencing.

## 2. Materials and Methods

### 2.1. Sample Collection of GC Patients

Ethics approval of this study was received from the Ethics Committee of Kunming Medical University. A total of 19 tumor tissues and 19 normal tissues were collected from a GC patient who underwent surgical resection. All patients in this study read and signed the informed consent. Preoperatively, the GC patient showed no pathological changes in other organs, and did not receive chemoradiotherapy and immunotherapy. The tissue was instantaneously frozen in liquid nitrogen after isolation until the experiment.

### 2.2. Cell Culture and Transfection

The human gastric cancer cell line MKN-45 and 293T/17 cells were purchased from Procell. GC cells were cultured in RPMI 1640 medium (Corning, USA), contained with 10% fetal bovine serum (FBS) and 1% penicillin/streptomycin (PS), in an incubator at 37°C containing 5% CO_2_. For gain-of-function, experiments, we designed a tRF-24-V29K9UV3IU sequence with a 5′ phosphate group (5′-P-UAGGAUGGGGUGUGAUAGGUGGCA-3′), and tRF-24-V29K9UV3IU mimics and control sequences were transfected into MKN-45 cells using Lipofectamine 2000 following the manufacturer's protocol. All sequences are shown in Supplemental Table 1.

### 2.3. Construction of a Stable Lentiviral Cell Line

The lentivirus-mediated tRF-24-V29K9UV3IU knockdown (LV-tRF-24-inhibitor sponge) vector and a negative control (NC) vector were purchased from Genepharma (China). The catalogue number of vector was C09004. In belief, the LV3(H1/GFP&Puro)-tRF-24-inhibitor sponge vector was constructed to express RNA sequences containing three mature tRF-24 binding sites, and the mature tRF-24 was bound by adsorption, thus exerting the inhibition effect of tRF-24. Virus packaging was performed using 293T tool cells, when cells reached 80–90% fusion, shuttle plasmid (LV3-tRF-24-inhibitor sponge) and packaging plasmid (pGag/Pol, pRev, pVSV-G) were added, and 300 *μ*L RNAi-mate was also added. The virus was collected after 72 h of culture. Then, when the confluence of MKN-45 cells reached 50%–70%, 50–200 *μ*L virus solution was added to each well and mixed. To find the optimal concentration of puromycin for selection, MKN-45 cells were treated with 0, 3, 6, 9, 12, and 15 *μ*g/mL puromycin (Selleck, China) for 1–4 days. To obtain stable transfections, MKN-45 cells with lentiviral infection of NC or LV-tRF-24-inhibitor sponge were cultured at optimal puromycin concentration for 14 days. The surviving cells were collected for subsequent experiments.

### 2.4. Xenografts in Mice

To establish the xenograft mouse model, 40 male BALB/c nude mice (5-week-old) were distributed at random into two groups (20 rats per group): a LV-tRF-24-inhibitor group and an NC group. For *in vivo* tumor growth assays (*n* = 5) [13], approximately 5 × 10^6^ MKN-45 cells were subcutaneously injected into the axilla of the BALB/c nude mice. Tumor volume (mm^3^) was calculated every 3 days with a caliper and calculated as (length × width^2^)/2. All mice were euthanized after 21 days using CO_2_ inhalation followed by decapitation. The collected tumor tissue was used for qRT-PCR and hematoxylin and eosin (H&E) staining. For *in vivo* tumor metastasis assays (*n* = 5) [14], approximately 2 × 10^6^ MKN-45 cells were injected into the tail veins of BALB/c nude mice. After 50 days, lung tissues from all BALB/c nude mice were subjected to H&E staining. All experiments in mice were approved by the Ethics Committee of Kunming Medical University.

### 2.5. H&E Staining

Tumor tissues (*n* = 3) and lung tissues (*n* = 5) from each group of mice were harvested for H&E staining to detect the tumorigenicity of tRF-24-V29K9UV3IU. Tumor and lung tissues were fixed in 10% formalin and paraffin embedded, then routine cut into 5 *μ*m sections, and mounted on slides. Following staining with H&E staining, tissues were imaged under a light microscope (Olympus, Japan).

### 2.6. qRT-PCR Analysis

TRIzol Reagent (Invitrogen, USA) was used to extract total RNA from tumor and adjacent peritumoral tissues of mice and then quality of RNA was assessed by NanoDrop 2000 (Thermo Scientific, USA). For tRF-24-V29K9UV3IU detection, to distinguish precursors from mature tRF, we used a two-end adaptor method for qRT-PCR. An adaptor was ligated at the 3′-ends of RNAs followed by hybridized with 3′ primers and ligated a 5′ adaptor. Finally, the product was amplified to create a cDNA using the Thermo Scientific revertaid first strand cDNA synthesis kit (Thermo Scientific, USA). For mRNA detection, conventional cDNA synthesis and PCR amplification were used. The specific PCR primers were designed and utilized to measure specific tRF and mRNAs by qRT-PCR using ABsolute Blue SYBR Green Master Mix (Thermo Scientific, USA) on the QuantStudio 6 Flex Real-Time PCR System (Thermo Fisher Scientific, Inc.) according to the product instructions. All primers used in this study were custom synthesized by Shanghai Sangon Biotech and are shown in Supplemental Table 1. The relative gene expression was normalized to *GAPDH* and determined by the 2^-*ΔΔ*Cq^ method [15].

### 2.7. Western Blotting

The method for western blotting was adapted from previously described [16]. Total protein was isolated from the tumors using RIPA lysis buffer and quantified using the BCA Protein Assay Kit (Thermo Scientific, USA). Next, equal amounts of protein were subjected to 10% SDS-PAGE, after that the proteins were transferred onto PVDF membranes. The PVDF membranes were blocked for nonspecific binding with 5% nonfat milk and incubated with primary antibodies: vimentin (1 : 1000, Abcam, ab8978), anti-Snail (1 : 1000, CST, 3879), and GAPDH (1 : 1000, Proteint, 60004-1-Lg), at 4°C overnight. Then, membranes were incubated with goat anti-mouse IgG H&L (HRP-conjugated secondary antibodies) (1: 10000, Abcam, ab205719) at room temperature for 1 h. In the end, the gels were photographed performed on the Bio-Rad ChemiDoc XRS system.

### 2.8. Transcriptome Sequencing and Bioinformatics Analysis

GC cells were harvested for transcriptome sequencing in triplicates after transfection with tRF-24-V29K9UV3IU mimics or NC. Total RNA was isolated using the TRIzol Reagent and qualified using the NanoDrop 2000 (Thermo Scientific, USA). Next, RNA was reversed transcribed and amplified into a cDNA library using the RNA Seq Library Preparation Kit for Transcriptome Discovery (Questgenomics, Nanjing, China), and RNA sequencing was performed on an Illumina HiSeq 2500 platform. The fragments per kilobase per million were used to normalize the expression of sequences, and the normalized expression was used to identify differentially expressed genes (DEGs) using the DEGSeq algorithm. The DEGs parameter was set to the absolute value of log_2_ (fold change, FC) was greater than 1 and *P* was less than 0.05. DEGs were used for GO classification and KEGG analysis.

### 2.9. Network Construction

To illustrate the regulatory network of tRF-24-V29K9UV3IU on the basis of target gene and KEGG analysis, we selected 8 DEGs and related pathways to perform the network. The network was centered on tRF-24-V29K9UV3IU, with target gene-mediated pathways, and it was imaged using the Cytoscape version 3.6.1.

### 2.10. Dual Luciferase Activity Assay

G-protein–coupled receptor 78 (GPR78) containing the wild-type (WT) or mutant (MUT) putative binding sites of tRF-24-V29K9UV3IU was synthesized by GenePharma (Shanghai, China) and cloned into psiCHECK-2 vectors (Promega, USA). Next, psiCHECK-2-GPR78-WT and psiCHECK-2-GPR78-MUT reporters were co-transfected into 293T/17 cells (CL-0469, Procell, China) with tRF-24-V29K9UV3IU mimics or controls (NC) using Lipofectamine 2000. After 48 h of transfection, luciferase activity was measured utilizing the Dual-Luciferase Reporter Assay System (Promega, USA). All sequences are shown in Supplemental Table 1.

### 2.11. Argonaute 2 (AGO2) and RNA Immunoprecipitation (RIP)

The RNA immunoprecipitation (RIP) assay was conducted by the Magna RIP™ RNA-Binding Protein Immunoprecipitation Kit (Cat. 17-701, Millipore, USA) according to the manufacturer's instructions. Shortly, cells were lysed in RIP lysis buffer (Magna RIP Kit, Millipore, MA, USA) and incubated with anti-pan-AGO antibodies (MABE56; Millipore), control IgG antibodies. Then, the RNA and proteins in the immunoprecipitates were harvested in the TRIzol Reagent or lysis buffer, respectively, for subsequent analysis.

### 2.12. CCK8 Assay

The proliferation ability of GC cells was assessed by CCK8 assays. Shortly, about 1 × 10^4^ cells were seeded into 96-well plates. Each group contained six replicate wells. Then, 10 *μ*L of CCK-8 (Beyotime Biotechnology) assay solution was mixed to each well and another cultured for 1 h. Finally, OD values at 450 nm were obtained using a microplate reader.

### 2.13. Transwell Migration and Invasion Assays

The Transwell system was used to assess the migration and invasion of GC cells. Transwell chambers (Corning, NY, USA) were covered with a layer of matrigel mix for invasion assays and not coated for migration assays. GC cells were seeded into the upper chamber and normal culture medium filled the bottom chamber. After incubating for 24 h, the upper chamber was removed, fixed, and stained with crystal violet (Beyotime Biotechnology). Cells were visualized and counted in five randomly selected fields under a microscope (×100).

### 2.14. Flow Cytometry Analysis

Cell apoptosis was accessed by flow cytometry; for this purpose, theharvested cell was gently washed by PBS and centrifuged with high-speed to remove the supernatant. Next, a cell suspension with a density approximately of 2–5 × 10^5^ cells/mL was precipitated using 195 *μ*L Binding Buffer and mixed with 5 *μ*L Annexin V-FITC solution (C1062, Beyotime, China) following incubated for 15 min at 25°C in the dark. Subsequently, cell mixture underwent centrifugation and re-suspension and washed by Binding Buffer. Finally, cells were incubated with 10 *μ*L propidium iodide. The stained cells were analyzed by FACSVerse™ (BD Biosciences, USA), and apoptosis data was processed using the Flowjo V10 software (Tree Star, San Francisco, CA, USA).

### 2.15. Statistical Analysis

Statistical analysis of all data was processed using SPSS 16.0 and significant differences between two groups were assessed by *t* test, one-way ANOVA followed by Tukey's test used for four groups. For all data, a *P* value less than 0.05 was deemed statistically significant. Data are presented as means ± SD.

## 3. Results

### 3.1. Knockdown of tRF-24-V29K9UV3IU Facilitates the Growth and Metastasis of Xenograft Tumors *In Vivo*

**Figure 1:**
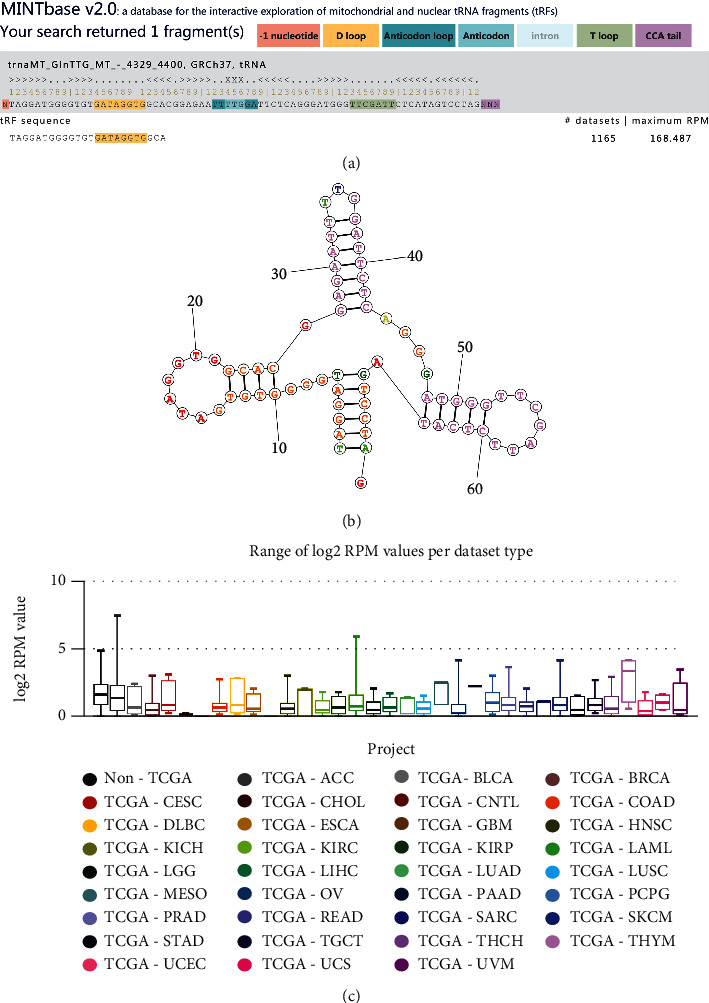
The information of tRF-24-V29K9UV3IU. (a) The sequence information of tRF-24-V29K9UV3IU, which was a 5′-tRF type cleaved from tRNA-Gln-TTG, in MINTbase v2.0. (b) Secondary structure of total tRNA-Gly. (c) The expression of tRF-24-V29K9UV3IU in different tumors predicted by TCGA.

The tRF-24-V29K9UV3IU was identified by small RNA sequencing in our previous study [12]. As shown in Figure 1(a), tRF-24-V29K9UV3IU (Sequence: TAGGATGGGGTGTGATAGGTGGCA) was a 5′-tRF type cleaved from tRNA-Gln-TTG, which was predicted by MINTbase v2.0 (a database for the interactive exploration of mitochondrial and nuclear tRFs, https://cm.jefferson.edu/). We also predicted the secondary structure of tRNA-Gln who produced tRF-24-V29K9UV3IU on the RNAstructure database (http://rna.urmc.rochester.edu/RNAstructureWeb/ Servers/Predict1/Predict1.html); it was exhibited a typically cloverleaf structure (Figure 1(b)). The Cancer Genome Atlas (TCGA) data showed that tRF-24-V29K9UV3IU was expressed in multiple tumor samples, and compared with non-TCGA, tRF-24-V29K9UV3IU was low expressed in most tumor tissues, including colon adenocarcinoma (COAD), esophageal carcinoma (ESCA), and stomach adenocarcinoma (STAD) (Figure 1(c)). In short, tRF-24-V29K9UV3IU is a novel small molecule that may be involved in GC progression.

**Figure 2:**
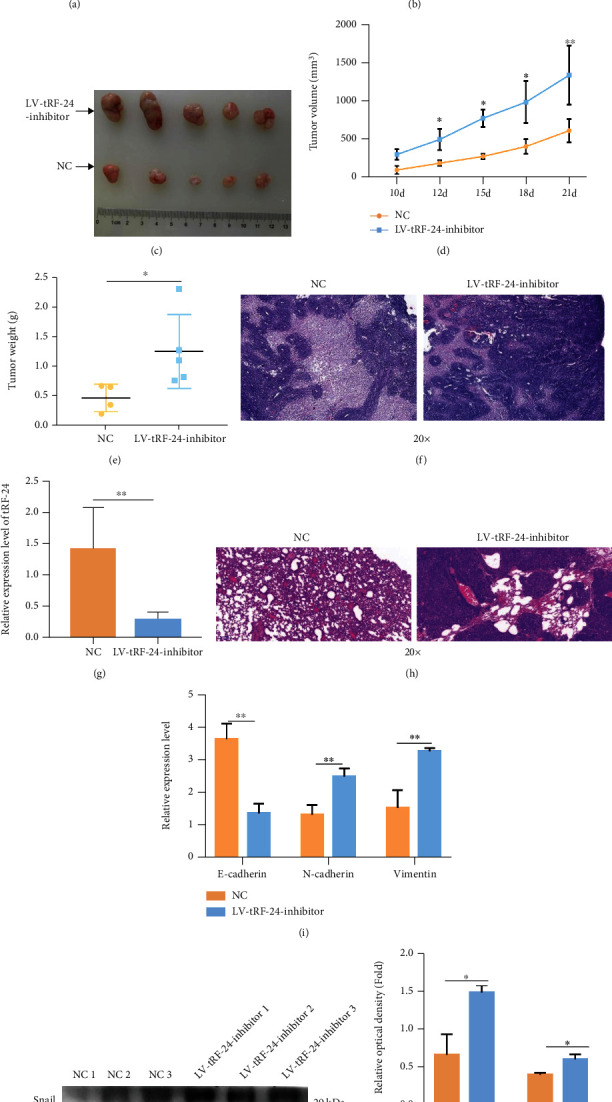
Knockdown of tRF-24-V29K9UV3IU facilitates growth and metastasis of xenograft tumors *in vivo.* (a) The efficacy of lentivirus interfering with tRF-24-V29K9UV3IU expression detected by qRT-PCR (*n* = 3). (b) Representative images of subcutaneous tumor formation in mice after LV-tRF-24-V29K9UV3IU-inhibitor-expressing or NC MKN-45 cell inoculation (*n* = 5). (c) Representative images of subcutaneous tumor tissues in mice (*n* = 5). The tumor (d) volume and (e) weight statistics after LV-tRF-24-V29K9UV3IU-inhibitor-expressing or NC MKN-45 cell inoculation (*n* = 5). (f) Representative images for H&E staining in tumors (*n* = 3). Magnification 20x. These results implicated that knockdown of tRF-24-V29K9UV3IU facilitates growth of GC. (g) The expression of tRF-24-V29K9UV3IU in tumor tissues after LV-tRF-24-V29K9UV3IU-inhibitor or NC MKN-45 cell inoculation (*n* = 3). (h) Representative images of H&E staining in lung tissue following LV-tRF-24-V29K9UV3IU-inhibitor-expressing MKN-45 cell injection (*n* = 5). Magnification 20x. (i) mRNA expression of E-cadherin, N-cadherin, and vimentin in tumor tissues after LV-tRF-24-V29K9UV3IU-inhibitor-expressing or NC MKN-45 cell inoculation, detected by qRT-PCR (*n* = 3). ((j) and (k)) Protein expression of vimentin and Snail in tumor tissues after LV-tRF-24-V29K9UV3IU-inhibitor-expressing or NC MKN-45 cell inoculation, detected by western blotting (*n* = 3). These results implicated that knockdown of tRF-24-V29K9UV3IU facilitates lung metastasis of GC. *t* test, ∗*P* < 0.05, ∗∗*P* < 0.01, ∗∗∗*P* < 0.001.

Next, to verify the function of tRF-24-V29K9UV3IU, lentiviral vectors were used to interfere with the expression of it. After 14 days of puromycin selection, we obtained an MKN-45 cell line with stable lentivirus-mediated knockdown of tRF-24-V29K9UV3IU, and the interference efficacy is shown in Figure 2(a). To observe the potential biological effects of tRF-24-V29K9UV3IU in GC tumor cells, MKN-45 cells stably transfected with LV-tRF-24-V29K9UV3IU-inhibitor sponge or NC vectors were injected subcutaneously into BALB/c nude mice (Figures 2(b) and 2(c)). Compared with the NC vector, knockdown of tRF-24-V29K9UV3IU significantly increased tumor growth, as determined by tumor weights and tumor volumes (Figures 2(d) and 2(e)). The tumor burden of mice gradually increased with time, but the volume of tumors in the LV-tRF-24-V29K9UV3IU-inhibitor group was always significantly bigger than in the NC group (Figure 2(d)). tRF-24-V29K9UV3IU knockdown-promoted tumor growth was confirmed by H&E staining (Figure 2(f)). We also detected the expression of tRF-24-V29K9UV3IU in tumor tissues; as expected, the larger the tumor volume, the lower the expression of tRF-24-V29K9UV3IU in the tumor (Figure 2(g)). Moreover, in the BALB/c nude mice, tail vein injection of MKN-45 cells stably transfecting LV-tRF-24-V29K9UV3IU-inhibitor sponge resulted in higher number of lung metastatic nodules than the injection of NC vector, as shown by H&E staining (Figure 2(h)). To further confirm the promotion of tumor metastasis after knocking down tRF-24-V29K9UV3IU, qRT-PCR and western blotting were performed for measuring the expression of epithelial–mesenchymal transition markers in tumor tissues. E-cadherin expression was decreased and the expression of N-cadherin and vimentin was up-regulated at the mRNA level, whereas Snail and vimentin was also up-regulated at the protein level, supporting that the knockdown of tRF-24-V29K9UV3IU promoted the epithelial–mesenchymal transition of tumor cells (Figures 2(i)–2(k)). These results suggested that tRF-24-V29K9UV3IU has a tumor suppressor role in tumorigenesis and aggressiveness.

### 3.2. Gene Expression Profile Alterations Triggered by tRF-24-V29K9UV3IU

**Figure 3:**
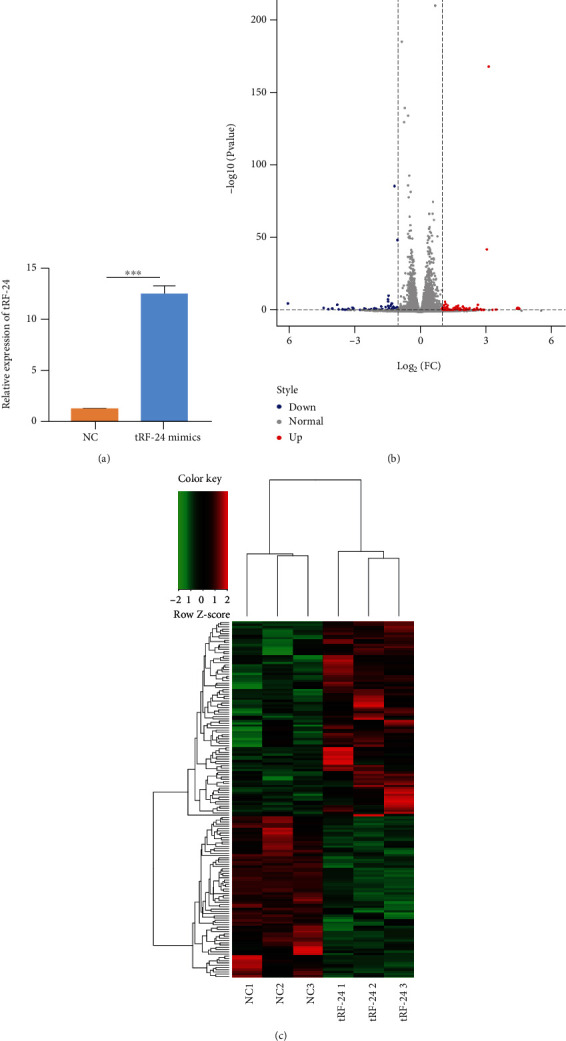
Gene expression profile after overexpression of tRF-24-V29K9UV3IU. (a) The efficacy of tRF-24-V29K9UV3IU overexpression by qRT-PCR. (b) Volcano plot of DEGs in MKN-45 cells after overexpression of tRF-24-V29K9UV3IU compared with the NC group. Red means up-regulated DEGs, and blue means down-regulated DEGs. (c) Heat map showing the DEGs in MKN-45 cells after the overexpression of tRF-24-V29K9UV3IU compared with the NC group. Red means up-regulated DEGs, and green means down-regulated DEGs. “tRF-24” indicates cells overexpressing tRF-24-V29K9UV3IU. *n* = 3, *t* test, ∗∗∗*P* < 0.001.

To take an overview of molecular alteration of tRF-24-V29K9UV3IU effect on GC cells, we first analyzed the gene expression profile overexpression of tRF-24-V29K9UV3IU by transfection with RNA mimics. Compared with the NC group, tRF-24-V29K9UV3IU was significantly overexpressed (Figure 3(a)). The overall gene expression profile alterations triggered by tRF-24-V29K9UV3IU is shown in Supplemental Table 2. Moreover, a total of 159 DEGs were identified, including 72 up-regulated and 87 down-regulated genes in the tRF-24-V29K9UV3IU-overexpressing MKN-45 cells relative to NC MKN-45 cells (Figure 3(b)). A heat map indicated significant dysregulation of mRNAs in the tRF-24-V29K9UV3IU-overexpressing MKN-45 cells compared with NC MKN-45 cells (Figure 3(c)). Therefore, we obtained the expression profile of GC cells after overexpression of tRF-24-V29K9UV3IU.

### 3.3. Functional and Pathway Analysis

**Figure 4:**
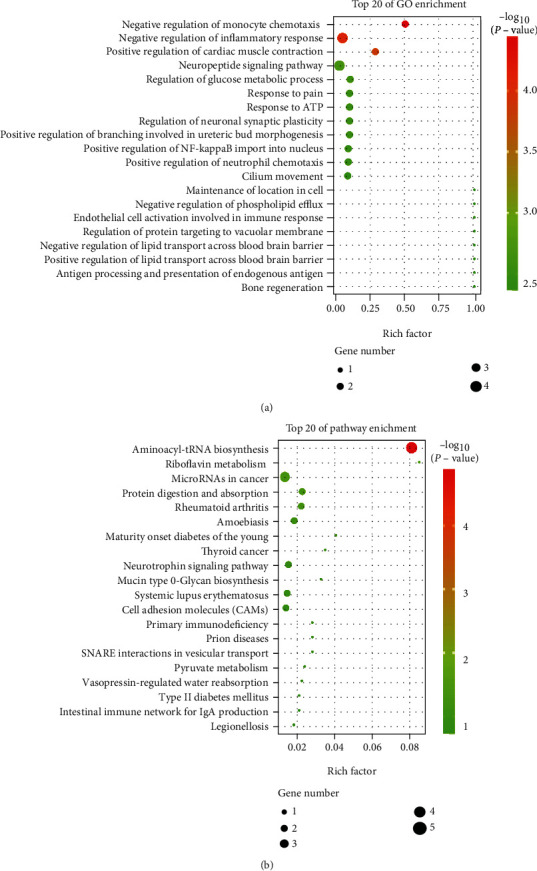
Functional analysis of DEGs. (a) Top 20 GO terms and (b) top 20 KEGG pathways identified among enriched DEGs. Circle size indicates the number of DEGs enriched, and red color indicates significant enrichment.

To further investigate the potential molecules that respond to tRF-24-V29K9UV3IU overexpression, all DEGs were annotated in the GO and KEGG databases. Functional GO analysis showed that these DEGs were mainly enriched in the negative regulation of monocyte chemotaxis, negative regulation of the inflammatory response, and positive regulation of NF-kappaB import into the nucleus (Figure 4(a)). KEGG analysis revealed that these DEGs were mainly involved in pathways associated with cancer and metastasis, such as microRNAs in cancer, thyroid cancer, and cell adhesion molecule pathways (Figure 4(b)). Therefore, we speculated that the tRF-24-V29K9UV3IU might regulate the GC progress by the above DEG-mediated pathways.

### 3.4. tRF-24-V29K9UV3IU Inhibits GPR78 Expression by Directly Binding to Its 3′-Untranslated Region (3′-UTR)

**Figure 5:**
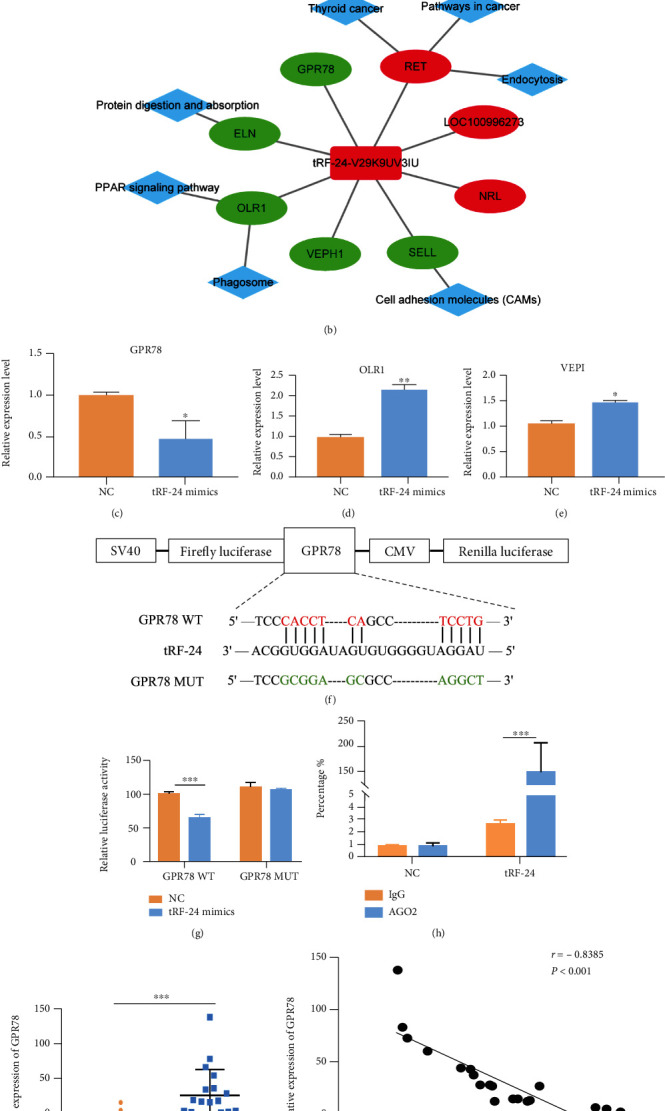
tRF-24-V29K9UV3IU inhibits GPR78 expression by binding its 3′-UTR in GC cells. (a) Eight DEGs predicted to possibly bind tRF-24-V29K9UV3IU through overlap of DEGs and the Miranda and RNAhybrid database. (b) The regulatory network of tRF-24-V29K9UV3IU. The rounded rectangle represents tRF-24-V29K9UV3IU, the ellipse represents mRNA, the diamond represents a pathway, and the red/green color indicates upregulation and down-regulation, respectively. ((c)–(e)) Expression of *GPR78*, *OLR1*, and *VEPH1* as verified by qRT-PCR. (f) Putative binding site of tRF-24-V29K9UV3IU on GPR78. (g) The luciferase activity of GPR78 in MKN-45 cells after cotransfection with tRF-24-V29K9UV3IU mimics or NC. (h) An AGO2-RIP assay was conducted to confirm that tRF-24-V29K9UV3IU associates with AGO2. (i) Expression of *GPR78* in 19 pairs tumor and normal tissues detected by qRT-PCR. Expression of *GPR78* was up-regulated in tumor compared to normal tissues. (j) The expression of *GPR78* and tRF-24-V29K9UV3IU was negatively correlated, which was analyzed by Pearson's correlation in tumors. The above experiments were repeated three times. *t* test, ∗*P* < 0.05, ∗∗*P* < 0.01, ∗∗∗*P* < 0.001.

Considering that tRF-24-V29K9UV3IU is down-regulated in tumor tissues, we focused on the DEGs predicted to be targets of tRF-24-V29K9UV3IU via overlap in the Miranda and RNAhybrid databases. There were a total of 8 DEGs that met these conditions and attracted our focus (Figure 5(a)). We constructed a network diagram to visualize the predicted regulatory network for tRF-24-V29K9UV3IU. The GPR78, ventricular zone expressed pH domain-containing 1 (VEPH1), and oxidized low-density lipoprotein (lectin-like) receptor 1 (OLR1), elastin (ELN), and selectin L (SELL) were down-regulation (green) in tRF-24-V29K9UV3IU-overexpressing MKN-45 cells compared with NC MKN-45 cells, whereas the three remaining predicted target genes were up-regulated (red) (Figure 5(b)). To verify whether the expression of these genes was suppressed in GC cells after tRF-24-V29K9UV3IU overexpression according to our transcriptome data, we selected three genes with the highest abundance for qRT-PCR validation (GPR78, VEPH1, and OLR1). Results showed that only down-regulation of GPR78 was confirmed in tRF-24-V29K9UV3IU-overexpressing MKN-45 cells compared with NC MKN-45 cells by qRT-PCR, while the expressions of VEPH1 and OLR1 were inconsistent with the RNA sequencing data (Figures 5(c)–5(e)). Therefore, GPR78 expression was selected as a downstream marker of tRF-24-V29K9UV3IU for subsequent experiments.

Next, we explored the interaction between tRF-24-V29K9UV3IU and GPR78. The full-length and mutant sequence of the 3′-UTR of wild-type GPR78 (GPR78-WT) and mutant GPR78 (GPR78-MUT) containing the predicted tRF-24-V29K9UV3IU binding sites was cloned into psiCHECK-2 vectors (Figure 5(f)). A luciferase reporter assay indicated that only the GPR78-WT plasmids significantly decreased luciferase activity in tRF-24-V29K9UV3IU mimics group, whereas there was no difference in the GPR78 mutants (Figure 5(g)). These results indicated that there might be a direct interaction between tRF-24-V29K9UV3IU and GPR78 at the 3′-UTR. Increasing articles have demonstrated that tRFs can bind to the Argonaute (AGO) complex and exert functions similar to miRNAs by complementarily binding to the 3′-UTRs of mRNAs [17, 18]. Therefore, we performed an AGO2-RIP assay in MKN-45 cells to pull down RNA transcripts bound to AGO2. We found that tRF-24-V29K9UV3IU was significantly enriched by AGO2-pulldown compared with the input control, implying molecular interaction between AGO2 and tRF-24-V29K9UV3IU (Figure 5(h)). In the previous study, we examined the expression of tRF-24-V29K9UV3IU in tumor tissues and normal tissues [12]. To interrogate whether the expression of GPR78 and tRF-24-V29K9UV3IU is correlated in tumor tissues, we examined GPR78 expression in 19 paired tumor tissues and normal tissues, followed by Pearson's analysis. The results showed that the expression of GPR78 in tumor tissues was strongly higher than that in normal tissues (Figure 5(i)), and GPR78 expression was significantly negatively correlated with tRF-24-V29K9UV3IU (*P* < 0.001) (Figure 5(j)). Cumulatively, tRF-24-V29K9UV3IU down-regulates GPR78 expression by binding to AGO2 and complementing with the 3′-UTR of GPR78.

### 3.5. tRF-24-V29K9UV3IU Inhibits the Biological Function of GC Cells by Regulating GPR78

**Figure 6:**
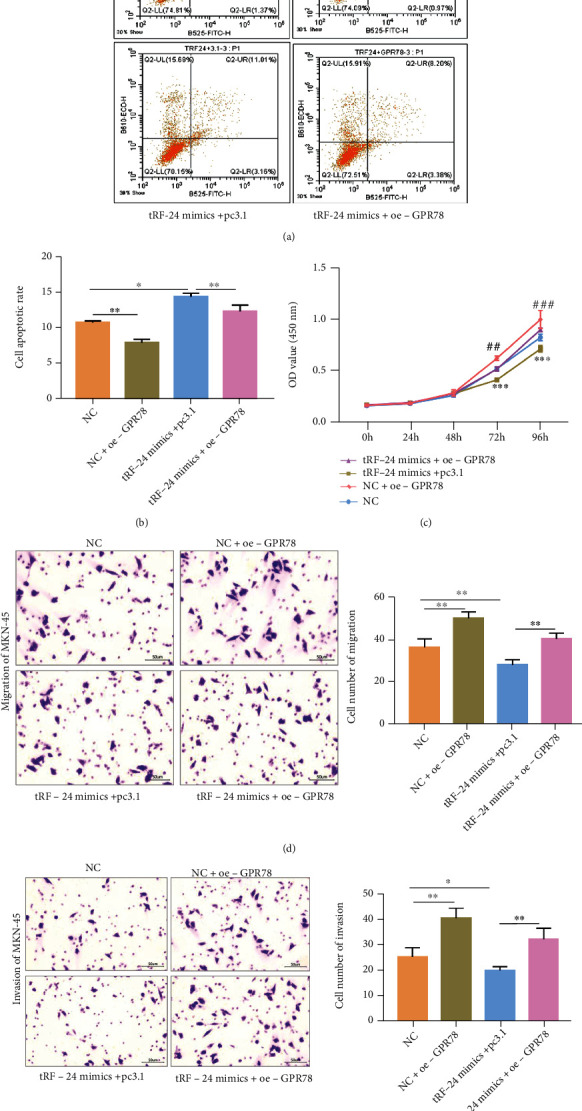
tRF-24-V29K9UV3IU inhibits the biological functions of GC cells by regulating GPR78 expression. (a) Representative images and (b) statistical results of flow cytometry analysis utilized to determine the number of apoptotic cells in four groups. (c) Cell proliferation assays of MKN-45 cells overexpressing tRF-24-V29K9UV3IU. (d) Transwell assays were used to detect the effect of overexpression of tRF-24-V29K9UV3IU on MKN-45 cell migration (d) and invasion (e). These results showed that overexpression of tRF-24-V29K9UV3IU promoted apoptosis, suppressed proliferation, migration, and invasion, while overexpression of BBB was opposite, and overexpression of GPR78 weakened the function of tRF-24-V29K9UV3IU. The above experiments were repeated three times. One-way ANOVA followed by Tukey's test, ∗*P* < 0.05, ∗∗*P* < 0.01.

To explore whether tRF-24-V29K9UV3IU exerts biological effects in GC cells via regulation of GPR78 expression, we conducted a series of rescue experiments. Flow cytometry results showed that the overexpression of tRF-24-V29K9UV3IU prominently increased apoptosis in MKN-45 cells; conversely, GPR78 overexpression significantly reduced the apoptosis of MKN-45 cells (Figures 6(a) and 6(b)). In addition, the cotransfection of tRF-24-V29K9UV3IU mimics and oe-GPR78 partially eliminated the effect on MKN-45 cells (Figures 6(a) and 6(b)). Similarly, growth curves suggested that overexpression of tRF-24-V29K9UV3IU prominently decreased the proliferative ability of MKN-45 cells, and GPR78 overexpression had the opposite effect, whereas the cotransfection of tRF-24-V29K9UV3IU mimics and oe-GPR78 counteracted each other (Figure 6(c)). Transwell assays implicated that the migratory and invasive capabilities of MKN-45 cells were significantly inhibited by tRF-24-V29K9UV3IU mimics but significantly enhanced by the overexpression of GPR78 (Figures 6(d) and 6(e)). Simultaneous action of tRF-24-V29K9UV3IU mimics and oe-GPR78 could partially dilute the effect (Figures 6(d) and 6(e)). Therefore, these results supported that tRF-24-V29K9UV3IU altered the biological functions of GC cells by down-regulating GPR78 expression.

## 4. Discussion

Accumulation studies prove that non-coding RNAs are widely involved in the occurrence and development of tumors [19]. Importantly, tRFs are expected to be an important target in cancer treatment owing to their role in tumor progression [20]. In this study, we provided evidences that the low expression of tRF-24-V29K9UV3IU in GC *in vivo* was related to the promotion of growth and metastasis of tumor cells. In addition, we found that tRF-24-V29K9UV3IU directly inhibited GPR78 expression to suppress the proliferation, invasion, and metastasis and promote apoptosis of GC cells.

Increasing studies have showed that tRFs serve a pivotal role in the development of tumors. For instance, tRFs (e.g., derived from the nuclear tRNA^Gly^ and tRNA^Leu^, the mitochondrial tRNA^Val^ and tRNA^Pro^) and isoforms of miRNAs (isomiRs) contributed to the race disparities in triplen-negative breast cancer [21]. Londin et al. reported the abundance profiles and biases in lengths of tRFs associated with metastatase and patient survival in uveal melanoma [22]. A novel mitochondrial tRF of i-tRF-Phe is a molecular prognostic biomarker in chronic lymphocytic leukemia [23]. The tRF-24-V29K9UV3IU is a mitochondrial 5′-tRF discovered in our previous work [12], and here, we demonstrate that it plays a negative regulatory role in GC. Also, we found that tRF-24-V29K9UV3IU is not only expressed in GC, but also lowly expressed in TCGA STAD and other TCGA COAD cancers. These results encourage us to believe that tRF-24-V29K9UV3IU plays a key role in the progression of GC and induces us to further explore its mechanism of function.

Accumulating evidence has demonstrated that tRFs have a functional mechanism similar to that of miRNAs. For example, tRNA-derived fragments of CU1276 can interact with AGO1-4 proteins, functioning just as miRNAs to modulate the proliferation and DNA damage response in B cell lymphoma [17]. Green et al. found that tRF-3003a was enriched in the AGO2/RNA-induced silencing complex, and the inhibitory effects of tRF-3003a on JAK3 were abolished by AGO2 knockdown [24]. 5′-tRF^His-GTG^ has also been identified as a miRNA-like small non-coding RNA whose function relied on AGO2 [25]. Thus, perhaps as reviewed by Venkates et al., tRFs usually masqueraded as miRNA [26]. We hypothesize that tRF-24-V29K9UV3IU also acts in this manner. Because the function of miRNAs depends on the AGO2 protein, we explored the relationship between tRF-24-V29K9UV3IU and AGO2, and AGO2–RIP revealed that the tRF-24-V29K9UV3IU was significantly enriched by AGO2 antibody rather than IgG. Therefore, tRF-24-V29K9UV3IU might bind to AGO2 and function as a miRNA-like RNA to regulate target gene activity and thus inhibit the progression of GC.

In addition, our results of transcriptome sequencing and luciferase assay show that GPR78 is physically bound and transcriptionally regulated by tRF-24-V29K9UV3IU. GPR78 belongs to the G-protein–coupled receptor superfamily, which is composed of approximately 800 different members, functioning as central nodes of many different signaling pathways participated in various aspects of human physiology [27]. The function of members of the GPR Class A orphan subfamily in regulating tumor progression has been widely reported. For instance, GPR48/LGR4 overexpression promoted thyroid tumor growth, lymph node metastasis, and recurrence *in vivo* and proliferation and migration of thyroid cancer cells *in vitro* [28]. The CXCL17-CXCR8 (GPR35) signaling axis promotes the proliferation and migration of breast cancer cells *in vitro* and *in vivo* [29]. Therefore, it is reasonable to speculate that GPR78 is involved in the regulation of tumor progression, and this has been partially confirmed. GRP78 promotes malignant phenotype of hepatocellular carcinoma by activating the Wnt/HOXB9 signaling pathway and chaperoning LRP6 [30]. GPR78 activates the G*α*q-Rho GTPase pathway to promote lung cancer cell migration and metastasis [31]. In this study, GPR78 was shown to significantly block the inhibition of proliferation, migration, and invasion of GC cells caused by the overexpression of tRF-24-V29K9UV3IU, suggesting that GPR78 promotes GC progression, but its activity is regulated by tRF-24-V29K9UV3IU. These results were consistent with those of the abovementioned studies. Therefore, we speculate that the inhibition effect of tRF-24-V29K9UV3IU on GC is achieved by physically regulating GPR78 expression.

Moreover, it is worth noting that in our previous studies, We found that tRF-24-V29K9UV3IU inhibited the biological function of GC cells [12], which was consistent with the results of this study. Also, using target and function prediction found that tRF target genes were major enriched in focal adhesion, stem cell differentiation regulation, cancer proteoglycan synthesis, Wnt, MAPK, and calcium signaling pathways. However, in this study, the pathway of KEGG enrichment was microRNAs in cancer, thyroid cancer, and cell adhesion molecule (CAM) pathways. The reason for this discrepancy may be related to the experimental design. In the previous study , the KEGG analysis was based on target gene prediction and enrichment of miRNA-like mechanism for all DEtRFs between tumor and adjacent tissues, whereas this study was performed enrichment for DEGs after tRF-24-V29K9UV3IU knockdown, these DEGs included those regulated by tRF-24-V29K9UV3IU through other mechanisms than miRNA-like. In summary, the analyzed tRF and the based regulatory mechanisms are different, so the enriched pathways will be different.

Interestingly, according to the results of MINTbase v2.0, tRF-24-V29K9UV3IU may be originated from mitochondrial tRNAs. Telonis et al. first reported the existence of multiple sequences in human mitochondrial tRNAs that are highly similar to human nuclear chromosomes, and called it mitochondrial tRNA-lookalikes [6, 32]. The plenty of mitochondrial tRNA-lookalikes, repetitive representation of tRNA templates in the nuclear genome, make it challenging to definitively determine the source of tRFs. Only a few specific and sensitive methods like MINTbase v2.0 can distinguish between mitochondrial tRFs and nuclear tRFs [33]. Thus, we tentatively believe that tRF-24-V29K9UV3IU originates from mitochondria. To date, a few studies have described the presence of mitochondrial tRFs and provided clue for their mitochondrial origin and function [34, 35]. Meseguer proposed two models of mitochondrial tRF biogenesis: 1) one of mitochondrial tRF translocated out of the mitochondria into cytoplasm where Dicer prepared them and then incorporated with AGO2 to regulate the expression of nuclear-encoded genes; 2) mitochondrial tRF processed by Dicer and loaded in AGO2 was occurred in the mitochondria, and then control the expression of mitochondrial DNA-encoded genes [36]. In our study, tRF-24-V29K9UV3IU incorporated with AGO2 and function as a miRNA-like RNA to regulate GPR78. Therefore, we speculate that the working mechanism of tRF-24-V29K9UV3IU is more plausible with the first model.

## 5. Conclusion

In summary, tRF-24-V29K9UV3IU inhibits growth and metastasis of GC *in vivo*. Moreover, tRF-24-V29K9UV3IU exerts a miRNA-like function and down-regulates GPR78, thereby inhibiting the proliferation, invasion, and migration as well as promoting apoptosis of GC cells *in vitro*. This study identifies novel targets and diagnostic biomarkers to develop molecular therapies for treatment of GC.

## Data Availability

The datasets used and/or analyzed in this study are available from the corresponding author upon reasonable request.

## Supplementary Materials

Supplementary 1 Supplemental Table 1. Primers information used in this study.

Supplementary 2 Supplemental Table 2. Clean data quality control and statistics.
