# Supplementary material for: The tRNA-Derived Fragment tRF-24-V29K9UV3IU Functions as a miRNA-like RNA to Prevent Gastric Cancer Progression by Inhibiting GPR78 Expression
Source: J Oncol. 2022 Apr 29;2022:8777697. doi: 10.1155/2022/8777697 (PMC9077451; doi:10.1155/2022/8777697)
Supplement: Supplementary 1 — Supplemental Table 1. Primers information used in this study. [file 8777697.f1.pdf]

Supplemental Table 1. Primers information used in this study.

Name	Sequences (5' – 3')
GAPDH-F	AGAAGGCTGGGGCTCATT
GAPDH-R	TGCTAAGCAGTTGGTGGTG
OLR1-F	TGCTTCACTCTCTCATTCTTAGC
OLR1-R	CACCATGGAGAGTAAAGAAACTG
GPR78-F	GGGTCTCCTGGTGATGGTAC
GPR78-R	TGTCCAGGAAGCCAATGACT
VEPH1-F	TGGAACATAACCTGAGACCCT
VEPH1-R	TCCTGCACAGTTCCTTGTTG
tRF-24 mimics	UAGGAUGGGGUGUGAUAGGUGGCA
	UGCCACCUAUCACACCCCAUCCUA
mimics NC	UUCUCCGAACGUGUCACGUTT
	ACGUGACACGUUCGGAGAATT
U6-F	CGATACAGAGAAGATTAGCATGGC
U6-R	ACGCTTCACGAATTTGCGT
tRF-24-F	CCGACGATCTAGGATGGGGT
tRF-24-R	CTCTTCCGATCTTGCCACCTAT
RNA 5' Adapter (RA5), part	GTTCAGAGTTCTACAGTCCGACGATC
RNA 3' Adapter (RA3), part	AGATCGGAAGAGCACACGTCT
Vimentin-R	AACTTACAGCTGGGCCATCG
Vimentin-F	AGTCCGCACATTCGAGCAAA
E-cadherin-F	GTCTCCTCTTGGCTCTGC
E-cadherin-R	CTGTCCTTTGTCGACCGGT
N-cadherin-F	GCCCAAGACAAAGAGACCCA
N-cadherin-R	TGGCCACTGTGCTTACTGAA
