# Supplementary material for: The tRNA-Derived Fragment tRF-24-V29K9UV3IU Functions as a miRNA-like RNA to Prevent Gastric Cancer Progression by Inhibiting GPR78 Expression
Source: J Oncol. 2022 Apr 29;2022:8777697. doi: 10.1155/2022/8777697 (PMC9077451; doi:10.1155/2022/8777697)
Supplement: Supplementary 2 — Supplemental Table 2. Clean data quality control and statistics. [file 8777697.f2.pdf]

Supplemental Table 2. Clean data quality control and statistics.

Samples	Total Reads Filter	Reads Filter (%)	Total Bases Filter	Bases Filter (%)	GC (%)	Mapped Rate (%)
NC1	20291818	0.952	3059446650	0.951	53.5	0.919
NC 2	29780738	0.954	4490417526	0.952	53.5	0.907
NC 3	30535674	0.949	4604137280	0.948	53.5	0.903
tRF24-1	37773348	0.941	5696351544	0.940	54.5	0.923
tRF24-2	20000744	0.937	3015099846	0.936	54.5	0.907
tRF24-3	43122062	0.936	6502386005	0.935	54.5	0.906
